# Supplementary material for: Pyruvate Dehydrogenase Kinase 1 inhibition mediated oxidative phosphorylation enhancement in cartilage promotes osteoarthritis progression
Source: BMC Musculoskelet Disord. 2023 Jul 20;24:597. doi: 10.1186/s12891-023-06585-6 (PMC10357736; doi:10.1186/s12891-023-06585-6)
Supplement: Supplementary file 1 — Additional file 1. [file 12891_2023_6585_MOESM1_ESM.zip › BP.Gsea.1653623667859/GOBP_NEGATIVE_REGULATION_OF_MIRNA_TRANSCRIPTION.html]

Details for gene set GOBP\_NEGATIVE\_REGULATION\_OF\_MIRNA\_TRANSCRIPTION[GSEA]

|  || Dataset | 55457\_collapsed\_to\_symbols.cls.cls #OA\_versus\_normal.cls.cls #OA\_versus\_normal\_repos |
| Phenotype | cls.cls#OA\_versus\_normal\_repos |
| Upregulated in class | normal |
| GeneSet | GOBP\_NEGATIVE\_REGULATION\_OF\_MIRNA\_TRANSCRIPTION |
| Enrichment Score (ES) | -0.59041244 |
| Normalized Enrichment Score (NES) | -1.7878512 |
| Nominal p-value | 0.0051457975 |
| FDR q-value | 0.10790664 |
| FWER p-Value | 0.987 |
Table: GSEA Results Summary

  

Fig 1: Enrichment plot: GOBP\_NEGATIVE\_REGULATION\_OF\_MIRNA\_TRANSCRIPTION      
 Profile of the Running ES Score & Positions of GeneSet Members on the Rank Ordered List

  

| SYMBOL | TITLE | RANK IN GENE LIST | RANK METRIC SCORE | RUNNING ES | CORE ENRICHMENT || 1 | SOX9 | SRY-box transcription factor 9 [Source:HGNC Symbol;Acc:HGNC:11204] | 2089 | 0.351 | -0.1142 | No |
| 2 | TGFB1 | transforming growth factor beta 1 [Source:HGNC Symbol;Acc:HGNC:11766] | 2490 | 0.303 | -0.1038 | No |
| 3 | NFATC3 | nuclear factor of activated T cells 3 [Source:HGNC Symbol;Acc:HGNC:7777] | 3079 | 0.242 | -0.1164 | No |
| 4 | RELA | "RELA proto-oncogene, NF-kB subunit [Source:HGNC Symbol;Acc:HGNC:9955]" | 3942 | 0.161 | -0.1613 | No |
| 5 | LILRB4 | leukocyte immunoglobulin like receptor B4 [Source:HGNC Symbol;Acc:HGNC:6608] | 3949 | 0.160 | -0.1399 | No |
| 6 | GATA6 | GATA binding protein 6 [Source:HGNC Symbol;Acc:HGNC:4174] | 5537 | 0.030 | -0.2590 | No |
| 7 | PPARG | peroxisome proliferator activated receptor gamma [Source:HGNC Symbol;Acc:HGNC:9236] | 6006 | -0.005 | -0.2947 | No |
| 8 | NFIB | nuclear factor I B [Source:HGNC Symbol;Acc:HGNC:7785] | 7729 | -0.136 | -0.4098 | No |
| 9 | BMP4 | bone morphogenetic protein 4 [Source:HGNC Symbol;Acc:HGNC:1071] | 7816 | -0.142 | -0.3971 | No |
| 10 | RARA | retinoic acid receptor alpha [Source:HGNC Symbol;Acc:HGNC:9864] | 10141 | -0.335 | -0.5317 | No |
| 11 | POU5F1 | POU class 5 homeobox 1 [Source:HGNC Symbol;Acc:HGNC:9221] | 10898 | -0.416 | -0.5336 | Yes |
| 12 | PPARA | peroxisome proliferator activated receptor alpha [Source:HGNC Symbol;Acc:HGNC:9232] | 11110 | -0.445 | -0.4892 | Yes |
| 13 | PPARD | peroxisome proliferator activated receptor delta [Source:HGNC Symbol;Acc:HGNC:9235] | 11301 | -0.471 | -0.4396 | Yes |
| 14 | NFATC4 | nuclear factor of activated T cells 4 [Source:HGNC Symbol;Acc:HGNC:7778] | 11363 | -0.481 | -0.3786 | Yes |
| 15 | YY1 | YY1 transcription factor [Source:HGNC Symbol;Acc:HGNC:12856] | 12229 | -0.653 | -0.3566 | Yes |
| 16 | REST | RE1 silencing transcription factor [Source:HGNC Symbol;Acc:HGNC:9966] | 12382 | -0.703 | -0.2723 | Yes |
| 17 | PDGFB | platelet derived growth factor subunit B [Source:HGNC Symbol;Acc:HGNC:8800] | 12389 | -0.704 | -0.1767 | Yes |
| 18 | SRF | serum response factor [Source:HGNC Symbol;Acc:HGNC:11291] | 12549 | -0.773 | -0.0834 | Yes |
| 19 | ZNF512B | zinc finger protein 512B [Source:HGNC Symbol;Acc:HGNC:29212] | 12623 | -0.809 | 0.0215 | Yes |
Table: GSEA details [plain text format]

  

Fig 2: GOBP\_NEGATIVE\_REGULATION\_OF\_MIRNA\_TRANSCRIPTION      
 Blue-Pink O' Gram in the Space of the Analyzed GeneSet

  

Fig 3: GOBP\_NEGATIVE\_REGULATION\_OF\_MIRNA\_TRANSCRIPTION: Random ES distribution      
 Gene set null distribution of ES for **GOBP\_NEGATIVE\_REGULATION\_OF\_MIRNA\_TRANSCRIPTION**
